# Different Gene Expression Patterns between Leaves and Flowers in *Lonicera japonica* Revealed by Transcriptome Analysis

**DOI:** 10.3389/fpls.2016.00637

**Published:** 2016-05-10

**Authors:** Libin Zhang, Yan Long, Chunhua Fu, Jun Xiang, Jianping Gan, Gang Wu, Haibo Jia, Longjiang Yu, Maoteng Li

**Affiliations:** ^1^Department of Biotechnology, College of Life Science and Technology, Huazhong University of Science and TechnologyWuhan, China; ^2^Hubei Collaborative Innovation Center for the Characteristic Resources Exploitation of Dabie Mountains, Huanggang Normal UniversityHuanggang, China; ^3^Institute of Biotechnology, Chinese Academy of Agricultural SciencesBeijing, China

**Keywords:** *Lonicera japonica*, transcriptome, transcription factors, differentially expressed genes (DEGs), network

## Abstract

The perennial and evergreen twining vine, *Lonicera japonica* is an important herbal medicine with great economic value. However, gene expression information for flowers and leaves of *L. japonica* remains elusive, which greatly impedes functional genomics research on this species. In this study, transcriptome profiles from leaves and flowers of *L. japonica* were examined using next-generation sequencing technology. A total of 239.41 million clean reads were used for *de novo* assembly with Trinity software, which generated 150,523 unigenes with N_50_ containing 947 bp. All the unigenes were annotated using Nr, SwissProt, COGs (Clusters of Orthologous Groups), GO (Gene Ontology), and KEGG (Kyoto Encyclopedia of Genes and Genomes) databases. A total of 35,327 differentially expressed genes (DEGs, *P* ≤ 0.05) between leaves and flowers were detected. Among them, a total of 6602 DEGs were assigned with important biological processes including “Metabolic process,” “Response to stimulus,” “Cellular process,” and etc. KEGG analysis showed that three possible enzymes involved in the biosynthesis of chlorogenic acid were up-regulated in flowers. Furthermore, the TF-based regulation network in *L. japonica* identified three differentially expressed transcription factors between leaves and flowers, suggesting distinct regulatory roles in *L. japonica*. Taken together, this study has provided a global picture of differential gene expression patterns between leaves and flowers in *L japonica*, providing a useful genomic resource that can also be used for functional genomics research on *L. japonica* in the future.

## Introduction

As a perennial, evergreen and twining vine, *Lonicera japonica* Thunb is widely cultivated in Asian countries such as China, Japan, and Korea (He et al., 2010). Pharmacological study has reported that *L. japonica* is used as a herbal medicine with anti-bacterial, anti-viral, anti-endotoxin, anti-inflammatory, and anti-pyretic effects (Hong et al., 1997). Therefore, *L. japonica* is often used to treat some human diseases including severe respiratory syndromes, H1N1 flu and hand-foot-and-mouth disease (Yuan et al., 2014a). *L. japonica* is also used as food, and worldwide as a healthy beverage, which recently led to the rapid increase in commercial value of Flos Lonicerae Japonicae (the *L. japonica* flower bud) in herbal medicine trading markets (Wang, 2010). Gene expression profiles of some important genes involved in chlorogenic acid and luteolin biosynthesis in *L. japonica* have been reported (Yuan et al., 2014b). Recently, next-generation sequencing (NGS) technology was used to examine transcriptome profiles of *L. japonica*. For example, there were 51,500 unigenes from *L. japonica* buds and leaves generated using the Roche/454 GS FLX platform (He et al., 2013). Also, over 32 million reads and over 6000 expressed sequence tags (ESTs) from *L. japonica* buds were obtained using the Illumina GAII platform (Illumina Inc., San Diego, CA, USA; Yuan et al., 2013). These studies discovered some important genes associated with the biosynthesis of active ingredients and provided an effective resource to study molecular genetics and functional genomics of *L. japonica* (He et al., 2013; Yuan et al., 2013, 2014a). NGS technology provides rapid and effective genome-wide transcriptome analysis and is now widely applied in many areas including biological, medical, and clinical drug development research. NGS technology provides a more sensitive and accurate way of analyzing transcriptome profiles, in contrast with microarray and other technologies (Schuster, 2008; Shendure and Ji, 2008; Wang Z. et al., 2009). Furthermore, NGS allows the discovery of novel and rare transcripts and the accurate quantification of gene expression (Cloonan et al., 2008; Shi et al., 2011; Tang et al., 2011; Yang et al., 2011). Various studies have reported that short sequencing reads have been successfully used for *de novo* genome and transcriptome assembly in organisms without genome reference, which greatly facilitated functional genomics study of many non-model plants (Knowles and McLysaght, 2009; Zhang et al., 2012; Marcolino-Gomes et al., 2013; Paritosh et al., 2013; Wang et al., 2013; Kim et al., 2014; Mudalkar et al., 2014; Zhou et al., 2014).

In this study, the transcriptome profiles of leaves and flowers in *L. japonica* were characterized by NGS. Over 200 million clean reads were generated using the Illumina Hiseq 2000 platform. *De novo* assembly of the *L. japonica* transcriptome was performed using the Trinity program (Grabherr et al., 2011). There were 150,523 unigenes obtained with an average length of 632 bp and an N_50_ of 947 bp. All unigenes were annotated with available protein and nucleotide databases. Furthermore, differentially expressed genes (DEGs) between leaves and flowers were identified and the three most important pathways were revealed by Gene Ontology (GO) and Kyoto Encyclopedia of Genes and Genomes (KEGG) analysis. Finally, multiple transcription factors (TFs) were detected in unigene libraries of leaves and flowers. Taken together, the transcriptome assembly and annotation of *L. japonica* tissues provide important genome information that will facilitate functional genomic research and metabolism regulation of medicinal composition in *L. japonica* in the future.

## Materials and methods

### Plant material and RNA isolation

The flowers and leaves of *L. japonica* were harvested and stored at −80°C. TRIzol (Invitrogen) was used to isolate total RNAs of the flowers and leaves according to the manufacturer's instructions. An Agilent 2100 Bioanalyzer (Agilent Technologies Inc.) was used to assess the integrity of the isolated total RNAs. DNase I (Promega) was then used to remove genomic DNA contamination. The isolated total RNAs were further quantified using a NanoDrop spectrophotometer (Thermo Fisher Scientific Inc.), and the purity of the total RNA was measured by calculating the A260/280 and A260/230 ratios. Finally, the purified RNA was dissolved in RNase-free water and stored in a −80°C freezer.

### cDNA preparation and sequencing

cDNA libraries were prepared using a TruSeqTM RNA sample preparation kit (Illumina) according to the manufacturer's instructions. In short, poly-A mRNA from total RNA of the flowers and leaves was purified using oligo (dT) magnetic beads (NEB). The collected mRNAs were first fragmented and further used to synthesize first-strand cDNAs with hexamer and reverse transcriptase (Promega). Subsequently, the second-strand cDNAs were synthesized with DNA polymerase I and RNase H. The obtained cDNA fragments were then purified, end-repaired, A-tailed and ligated to index adapters (Illumina). The ligation products were amplified by PCR and sequenced using the Illumina HiSeq2000 platform and a 100-bp pair-end sequencing protocol was employed.

### *De novo* transcriptome assembly

*De novo* assembly of the unigenes in *L. japonica* was performed as described by Zhang et al. (2013). Briefly, Illumina Pipeline software was first used to transform the raw image data generated into sequence information after the cDNA libraries were sequenced. The clean reads were generated by removing adaptor sequences and low-quality reads and then deposited in NCBI Sequence Read Archive (SRA) Sequence Database with accession number (SRP052594). The Trinity program was used to assemble the clean reads and obtain non-redundant unigenes (Grabherr et al., 2011). In short, reads which overlapped were assembled to generate contigs. The generated contigs were joined into scaffolds which were further assembled through gap filling to generate unigenes. In this study, default k-mer size of 25 was set for the *de novo* transcriptome assembly of the flowers and leaves. All other parameters were set as default values and the length of the assembled unigenes used for further study was ≥200 bp.

### Transcriptome annotation

Annotation of the assembled unigenes was performed by searching transcripts against the NCBI non-redundant protein (Nr), COG (Cluster of Orthologous Groups), Swiss-Prot, and Trembl databases. The search was conducted using BLASTx with an *E*-value cut-off of 1e^−5^. According to the BLAST hits identified by interrogation of the Nr and Swiss-Prot databases, the annotation program BLAST2GO was used to obtain cellular component, molecular function, and biological process terms (Conesa et al., 2005). The KEGG metabolic pathway database (http://david.abcc.ncifcrf.gov/) was used to annotate the pathway of these unigenes.

### Identification of DEGs

The RPKM (Reads Per kb per Million reads) of each unigene in leaf and flower tissues was calculated and used to measure gene expression level as described by Mortazavi et al. (2008). The edgeR program was used to determine the DEGs with a log-fold expression change (log FC) > 2 or < −2 using a threshold of false discovery rates (FDR < 0.001) and a high significance value (*P* < 0.005).

### qRT-PCR validation of DEGs

mRNA expression levels in the leaf and flower tissues of *L. japonica* were determined by quantitative reverse transcription-PCR (qRT-PCR) assay. Of total RNA, 1 μg was reverse-transcribed using SuperScript III Reverse Transcriptase (Invitrogen) and oligo (dT) 18 according to the manufacturer's protocol. The qPCR experiment was carried out using an ABI 7300 Real-Time PCR System (ABI) and each reaction was performed in triplicate. For mRNA expression detection, U6 RNA was set as an internal reference gene. The primers for qPCR are listed in Supplementary Dataset 1.

### Functional enrichment analysis

The GO functional enrichment analysis of down- and up-regulated DEGs in the network was performed by Bingo plugin in Cytoscape software (Maere et al., 2005). TF-based network analysis was constructed using the plugin of Agilent Literature Search in Cytoscape software.

### TF identification in *L. japonica*

All assembled unigenes were searched against the plant TF database (PlnTFDB; http://plntfdb.bio.uni-potsdam.de/v3.0/downloads.php) to identify TFs using BLASTx (cut-off *E*-value of 1e^−5^; Kalra et al., 2013).

## Results

### Sequencing and *de novo* transcriptome assembly of *L. japonica*

NGS technology has significantly promoted multiple functional genomics studies in many non-model plants that do not yet have a reference genome. To obtain transcriptome expression profiles in *L. japonica*, flowers and leaves were used for transcriptome sequencing and analysis. Two cDNA libraries were constructed from the total RNA of fresh flowers and leaves. The libraries were sequenced using the Illumina Hi-Seq2000 platform, and a total of 239,405,270 clean pair-end reads (including 124,617,394 reads in flowers and 114,787,876 in leaves) were obtained after removing adaptor sequences and low-quality reads. The Trinity program was then used for *de novo* assembly of all clean reads, which generated a total of 150,523 unigenes with an average length of 632 bp and an N_50_ of 947 bp (Table 1). Among these unigenes, the shortest and longest were 201 and 16,473 bp, respectively. The length distribution of the unigenes was also investigated. There were 83,664 unigenes in the range of 200–400 bp, 43,027 within 400–1000 bp and 21,015 within 1000–3000 bp (Figure S1). We also found 2817 unigenes of >3000 bp, which will be useful for further annotation and functional analysis.

**Table 1:** **Assembly summary of flower and leaf unigenes of ***L. japonica*****.

**Assembly**	**Number**
Number of clean reads (Leaf)	124,617,394
Number of clean reads (flower)	114,787,876
Total unigenes generated	150,523
N_50_ length (bp)	947
Average unigene length (bp)	632

### Annotation of the *L. japonica* transcriptome

**Figure 1:**
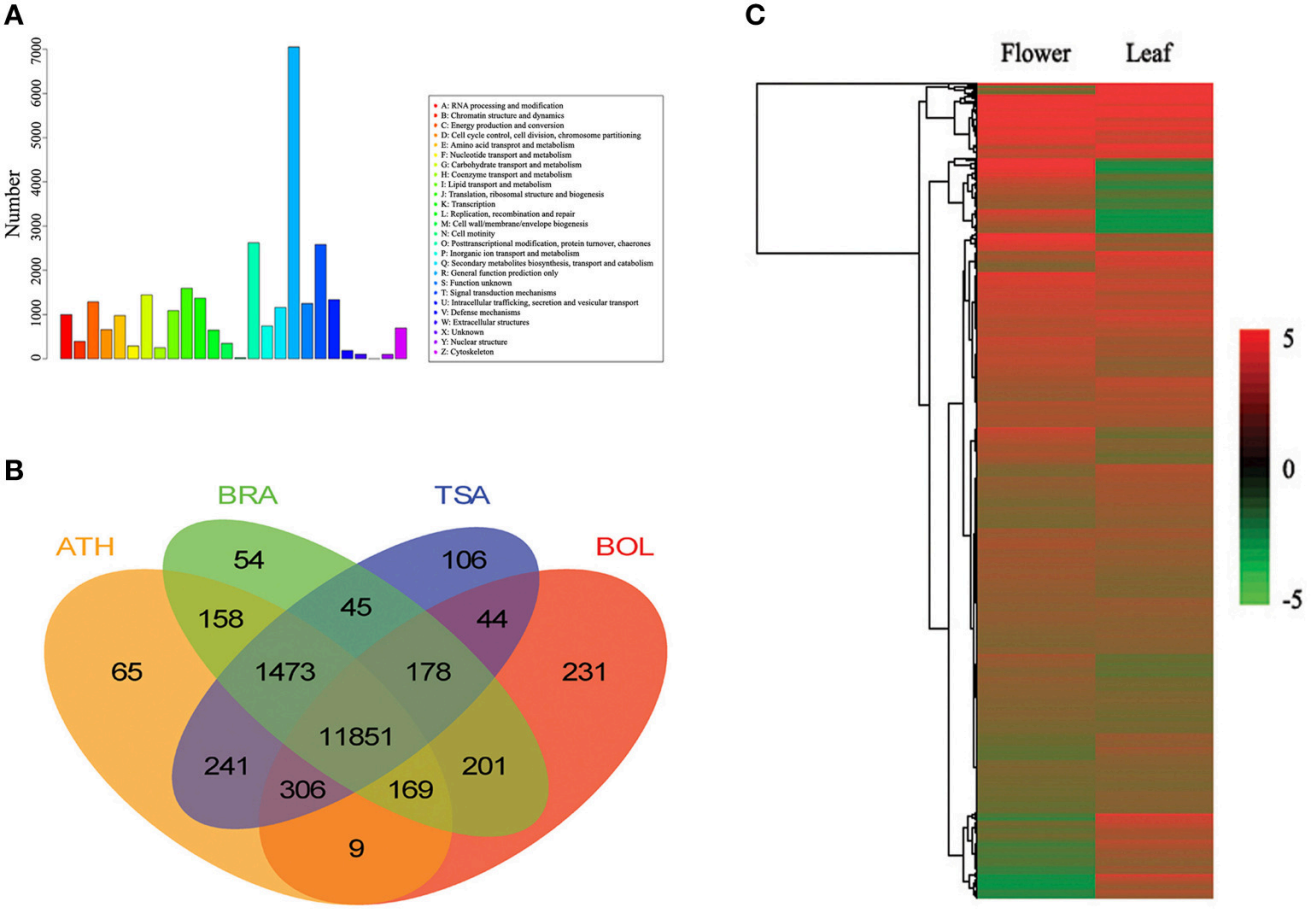
**Transcriptome annotation and expression analysis of ***L. japonica*** unigenes. (A)** Transcriptome annotation of *L. japonica* unigenes against COGs database. **(B)** Mapping result of *L. japonica* unigenes to familiar species. The homologs of *L. japonica* unigenes in various plant species [including *Arabidopsis thaliana* (ATH), *Thellungiella salsuginea* (TSA), *Brassica rapa* (BRA) and *Brassica oleracea* (BOL)] were shown. **(C)** Heatmap analysis of top 10,000 highly-expressed unigenes both in flower and leaf tissues.

The assembled unigenes of flowers and leaves in *L. japonica* generated by the Trinity program were annotated using BLASTx similarity analysis (*E* ≤ 1e^−5^) of common protein databases including the NCBI COG (Figure 1A), NCBI Nr and Swiss-Prot databases. Approximately 32.7, 29.1, and 15.5% of unigenes were mapped to Nr, Swiss-Prot, and COG, respectively (Table 2). There were 23,331 unigenes assigned to COG classifications (Figure 1A). Among the 25 COG categories, the cluster for “general function prediction only” (7050, 30.2%) was the largest group; followed by “posttranslational modification, protein turnover, chaperones” (2621, 11.2%); “signal transduction mechanisms” (2584, 11.1%); and “translation, ribosomal structure, and biogenesis” (1593, 6.9%).

**Table 2:** **Blast results of ***L. japonica*** unigenes**.

**Database**	**Total unigenes**	**Mapped unigenes**
Nr	150,523	49, 247 (32.72%)
SwissProt		43, 829 (29.12%)
COGs		26, 326 (17.49%)

Importantly, 32.7% of the unigenes were mapped to the Nr library, suggesting that many of the unigenes could be translated into proteins. Distribution analysis based on BLASTx searches showed that the unigenes of *L. japonica* had homologs in numerous plant species (Figure 1B). Among various plant species, the unigenes of *L. japonica* had the highest number of hits compared to sequences from *Arabidopsis thaliana* (54.94%), *Thellungiella salsuginea* (54.83%), *Brassica rapa* (54.39%), and *B. oleracea* (50%). As *L. japonica* is a type of vine, we performed a distribution analysis among *L. japonica, Vitis vinifera, Populus euphratica*, and *P. trichocarpa* Torr. & Gray. The result showed that *L. japonica* had 25,353 homologous genes with *V. vinifera, P. euphratica* and *P. trichocarpa*; and *L. japonica* had more hits when compared with *P. euphratica* (62.3%), *P. trichocarpa* (62.1%), and *V. vinifera* (54.2%; Figure S2).

### Different gene expression between flowers and leaves in *L. japonica*

**Figure 2:**
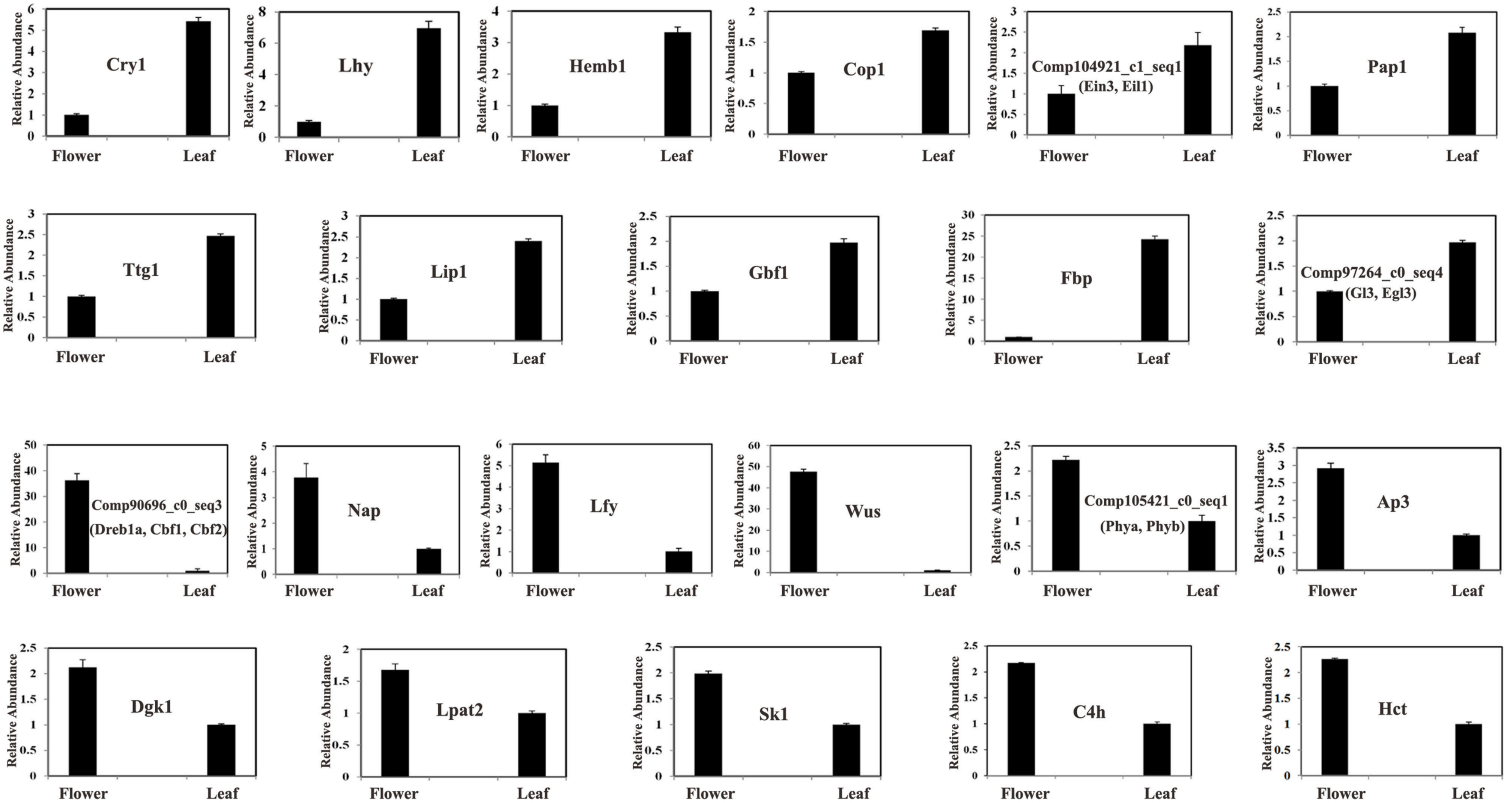
**Quantitative RT-PCR Validation of differentially expressed unigenes between leaf and flower tissues in ***L. japonica*****.

There were 86,510 and 129,764 unigenes identified in flowers and leaves, of which 65,751 were observed in both flowers and leaves. Among them, the top 10,000 highly-expressed unigenes both in flower and leaf tissues were displayed by heatmap analysis (Figure 1C). In order to identify DEGs between flowers and leaves, we set the expression level of unigenes in flower as a control and detected the up- or down-regulated unigenes in leaves. The results indicated that 35,327 unigenes were differentially expressed between leaf and flower tissues: 26,680 up-regulated and 8647 down-regulated (FDR ≤ 0.001, Supplementary Datasets 2, 3). Furthermore, we randomly selected 25 differentially expressed unigenes for qRT-PCR analysis. The mRNA expression level of 22 differentially expressed unigenes (11 up-regulated and 11 down-regulated) were confirmed by qRT-PCR (Figure 2). For example, TFs comp104921_c1_seq1 (Ein3, Eil1) and Gbf1 were up-regulated in leaves, while Nap1 was down-regulated (Figure 2). In addition, there were 20,759 unigenes detected only in flowers, and 64,013 only expressed in leaves. These DEGs between leaves and flowers were further annotated with GO terms that were classified into three categories: biological process, cellular component, and molecular function. These categories contained 13,893, 11,104, and 8110 DEGs, respectively (Figure S3).

**Figure 3:**
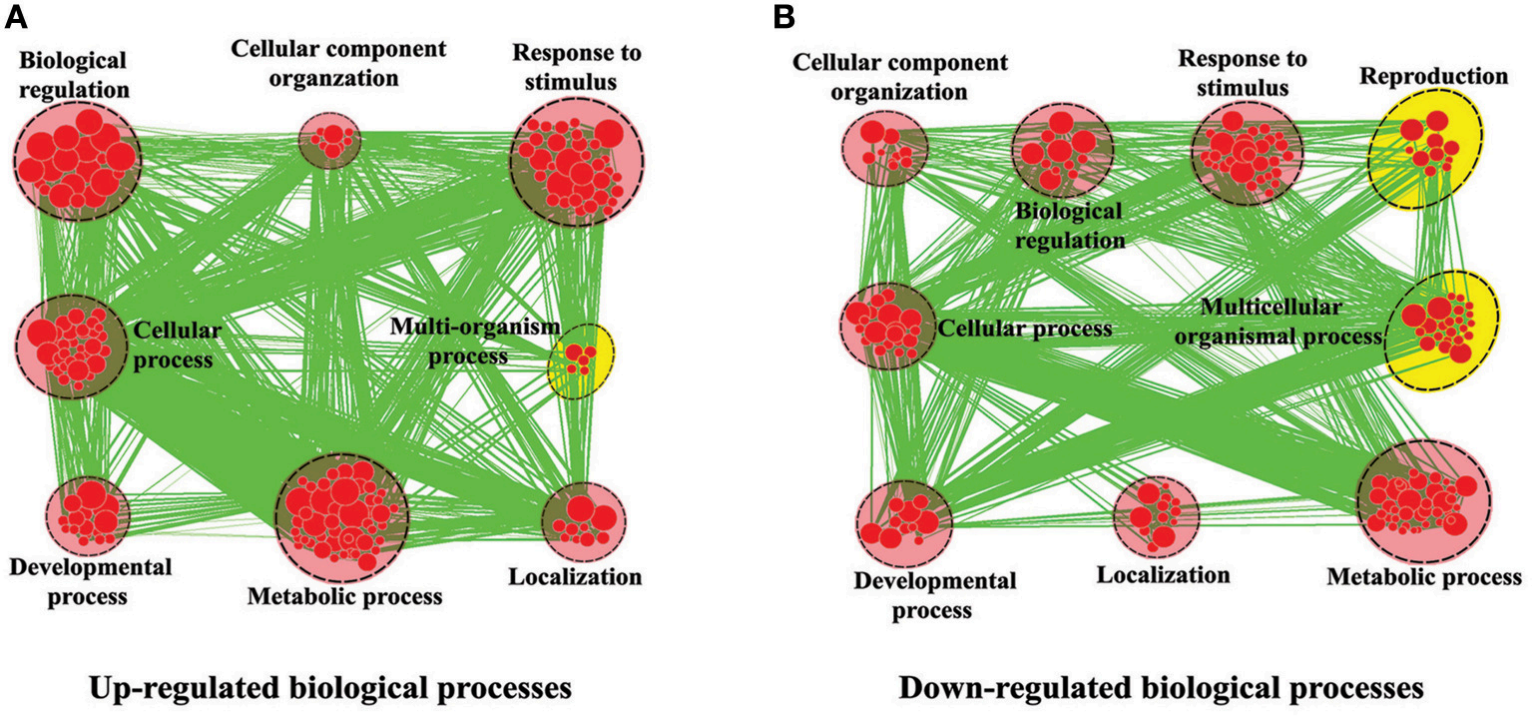
**Biological Process analysis of DEGs between leaf and flower tissues**. GO modules enriched with up-regulated DEGs **(A)** and down-regulated DEGs **(B)** were visualized by the Enrichment Map in Cytoscape. The red and yellow circles indicate the common and different biological processes between up-regulated and down-regulated DEGs, respectively.

For functional annotation of DEGs, the DEGs were also analyzed using Cytoscape Enrichment Map (http://www.cytoscape.org/). There were 6602 DEGs assigned with Biological Process terms (Figure 3). The common terms between up-regulated (Figure 3A) and down-regulated genes (Figure 3B) were “Metabolic process,” “Response to stimulus,” “Cellular process,” “Cellular component organization,” “Biological regulation,” “Localization,” and “Developmental process.” Furthermore, some DEGs were clustered in different GO terms. For example, within up-regulated genes, 35 (0.5%) genes were significantly enriched in “Multi-organism process.” Nevertheless, within the down-regulated genes, 297 (4.5%) genes were significantly enriched in “Multicellular organismal process” and 129 (2.0%) genes were significantly enriched in “Reproduction.” Among these genes, AT5G66570 encodes a 23-kDa extrinsic protein, called OEE2 (Oxygen-evolving enhancer protein). OEE2 is a component of photosystem II and participates in the regulation of oxygen evolution. Moreover, the phosphorylation of OEE2 is dependent on calcium. Collectively, OEE2 up-regulation in leaf tissue (Figure S4) is very consistent with the biological functions of leaf tissues and supports the feasibility of GO classifications in Figure 3.

### KEGG pathway analysis in *L. japonica*

**Figure 4:**
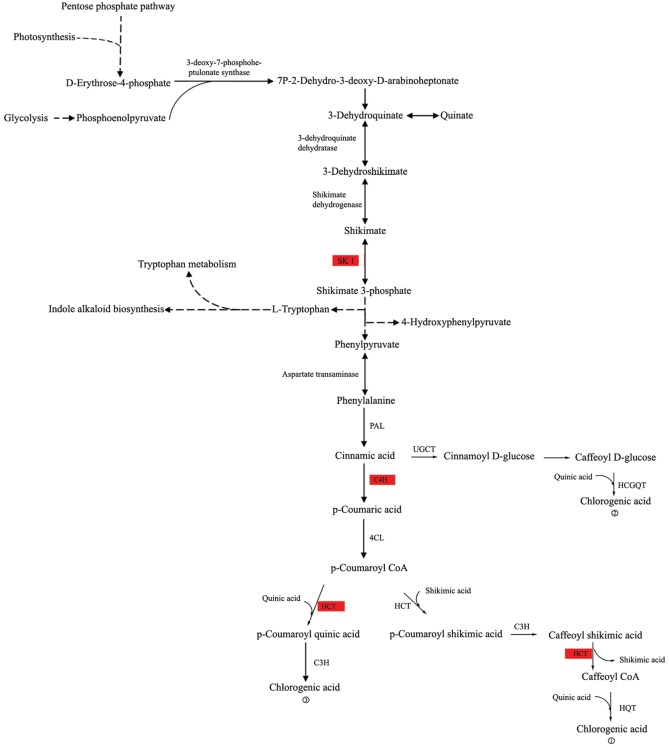
**The sketch map of the synthesis pathway of chlorogenic acid in ***L. japonica*****. The red rectangles indicate up-regulated genes including SK1, C4H, and HCT genes.

To better understand the biological functions of assembled unigenes in *L. japonica*, the DEGs between flowers and leaves were further assigned to the biochemical pathway analysis in the KEGG database (Kanehisa and Goto, 2000). As shown in Supplementary Dataset 4, 564 unigenes were assigned to the top 10 KEGG biochemical pathways (ranked by *P*-value). The top three pathways included “Ribosome” (198 unigenes, *P* = 1.67 × 10^−9^), “Oxidative phosphorylation” (109 unigenes, *P* = 1.46 × 10^−6^), and “Glycolysis/gluconeogenesis” (47 unigenes, *P* = 2.64 × 10^−6^). This result suggested that the DEGs were associated with the growth and metabolism activities of flower and leaf tissues in *L. japonica*. Among them, ribosomes are found in all cellular organisms and are responsible for synthesis of proteins in cells, while oxidative phosphorylation is the metabolic pathway in which mitochondria in cells use the enzymes and energy released by the oxidation of nutrients to synthesize ATP. For example, many other DEGs were involved in the process of “Glycolysis/gluconeogenesis” (Figure S5). As an energy-conversion process, glycolysis/gluconeogenesis is associated with the photosynthesis pathway. For instance, glucose is one of the main products of photosynthesis and fuels for many biological activities, while glycolysis is a metabolic pathway that converts glucose into pyruvate, in which the released free energy is used to synthesize ATP compounds. Our result showed that many genes (Figure S5, red column) involved in the “Glycolysis/gluconeogenesis” pathway were up-regulated in flowers of *L. japonica*, such as FBP, mtLPD1, and HCEF1. FBP encodes a fructose-1,6-bisphosphatase and catalyzes the formation of fructose-6-phosphate for sucrose biosynthesis. Collectively, these results suggest that DEGs have important regulatory roles in glycolysis/gluconeogenesis. We also observed that many DEGs were involved in the pathway of glycerophospholipid metabolism (Figure S6). For example, PSD1 (phosphatidyl decarboxylase 1) was up-regulated in flower tissues, while DGK1 (diacylglycerol kinase1) and LPAT2 (lysophosphatidyl acyltransferase 2) were down-regulated in flower tissues. Importantly, various studies have shown that chlorogenic acid is a primary active component in *L. japonica* and has multiple properties including anti-oxidant (Tsuchiya et al., 1996), anti-inflammatory (Lee et al., 1995), anti-carcinogenic (Mori et al., 1986), and anti-viral effects (Wang G. F. et al., 2009). In this study, three important genes involved in the synthesis pathway of chlorogenic acid, such as SK1 (shikimate kinase 1), C4H (cinnamate 4-hydroxylase), and HCT (hydroxycinnamoyl-coenzyme A shikimate/quinate hydroxycinnamoyltransferase), were up-regulated in flowers (Figure 4). This result showed that *L. japonica* flowers contained a higher proportion of chlorogenic acid than leaves, suggesting that chlorogenic acid synthesis was more active in flowers than leaves.

### TF identification in the *L. japonica* transcriptome

**Figure 5:**
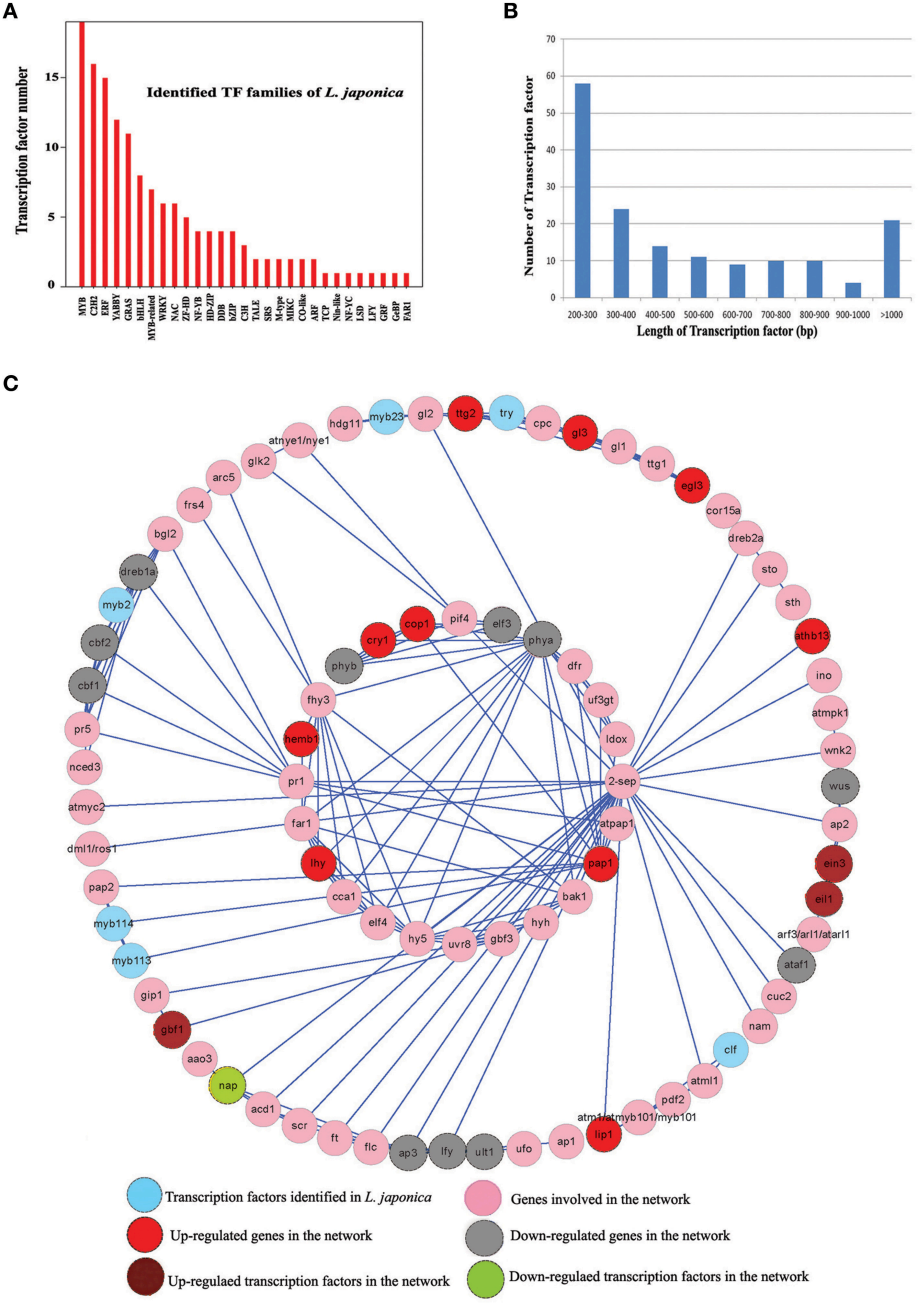
**Discovery of the transcription factors (TFs) and Construction of TFs-based regulation network in ***L. japonica***. (A)** Distribution of identified transcription factors in various TFs families. **(B)** Length distribution of the identified transcription factor genes. **(C)** Construction of TFs-based regulation network in *L. japonica* using Cytoscape software.

The assembled unigenes in flowers and leaves were subjected to BLASTx similarity analysis (*E* ≤ 1e^−5^) against the PlnTFDB, and 144 potential TFs were identified (Figure 5A). The length of these TF unigenes varied from 209 to 1512 bp, with an average value of 550 bp and an N_50_ of 396 bp. The 200–300-bp class was the most enriched in total sequence number (36.02%), followed by 300–400 bp (14.91%), >1000 bp (20.21%), 400–500 bp (8.69%), 500–600 bp (6.83%), 700–800 bp (6.21%), 800–900 bp (2.83%), 600–700 bp (5.59%), and 900–1000 bp (3.08%; Figure 5B). The potential TFs were distributed in 29 families (Figure 5A and Supplementary Dataset 5). Among them, the MYB family contained 19 TFs, followed by C2H2 (16), ERF (15), YABBY (12), and GRAS (11). For example, the YABBY family possesses a characteristic zinc finger domain close to the N-terminal end and a helix-loop-helix “YABBY” domain close to the C-terminal end (Bowman, 2000).

Cytoscape software was used to construct a TF-based regulation network between TFs and their gene targets. There were 84 nodes and 171 edges obtained in *L. japonica*. A total of 10 TFs, such as the MYB family, ein3, eil1, gbf1, and nap, were involved in the complicated regulation network (Figure 5C). Among them, DEG analysis showed that ein3, eil1, and gbf1 were up-regulated in leaves compared with flowers, while *nap* was down-regulated in leaves. Ein3 and its closest homolog eil1 are two primary TFs downstream of ein2 and are very important for the induction of multiple ethylene response genes (Alonso et al., 2003; An et al., 2010). Furthermore, Li et al. (2013) found that ein3 was a senescence-associated TF and accelerated age-dependent leaf senescence by directly inhibiting the transcription level of miR164 in *Arabidopsis*. In the present study, network analysis showed that ein3 interacted with two targets including eil1 and ap2. Both eil1 and ap2 were linked with the ethylene-mediated signaling pathway. Therefore, the results indicate that ein3, eil1, and ap2 may form a complex gene network to regulate the process of leaf senescence in *L. japonica*. Furthermore, we observed that ein3 was down-regulated in flowers, suggesting that there was a different signal pathway in the regulatory process of flower senescence in *L. japonica*. Meanwhile, we observed that ap3 interacted with nap in the network; and ap3 was also down-regulated in leaf tissue (Figures 2, 5C), suggesting that nap positively regulated the expression level of ap3. Finally, all genes involved in this network were overlapped with DEGs between flower and leaf tissues. The result showed that 12 genes (including one TF) and 13 genes (including three TFs) in this network were down- and up-regulated in leaves, respectively (Figure 5C).

## Discussion

*L. japonica* is a woody perennial, evergreen to semi-evergreen vine. The dried leaves and flowers are employed in traditional Chinese medicine to treat fever, headache, cough, thirst and sore throat. Components isolated from *L. japonica* include phenolic acids and flavonoids, which have been reported to have pharmacological functions (Tang, 2008). However, due to the limited genome information of *L. japonica*, functional genomic studies and gene discovery are greatly limited in *L. japonica*. NGS technologies have provided powerful tools for high-throughput sequencing, allowing easy discovery of novel genes by obtaining massive sequence information with enormous depth and coverage. For example, Li et al. (2015) obtained a total of 62,348,602 clean reads using the Illumina sequencing platform, which generated 66,026 unigenes in *Sophora moorcroftiana* by use of the Trinity program. They identified a large number of important genes involved in drought tolerance.

In the present study, we sequenced two cDNA libraries from *L. japonica* leaves and flowers using the Illumina Hiseq 2000 platform and obtained 150,523 unigenes. Notably, more unigenes were identified in this study than identified in previous work; for instance, Yuan et al. (2013) obtained over 32 million reads and over 6000 ESTs from a library made from *L. japonica* buds. Also, He et al. (2013) obtained 51,500 unigenes from *L. japonica* buds and leaves using the Roche/454 GS FLX platform. Nevertheless, we observed that < 50% of the unigenes were successfully annotated by BLAST against public databases including Nr, Swiss-Prot and COG. The possible cause of this result is the absence of genome information of *L. japonica* and technical limitations such as sequencing depth and read length. Gene expression profiles in different tissue types have been extensively studied to reveal tissue-specific gene functions. For example, based on microarray technology, Kram et al. (2009) identified many DEGs between secretory lateral nectaries and non-secretory median nectary tissues, as well as between mature lateral nectaries (post-anthesis) and immature lateral nectaries (pre-anthesis), which provided a promising approach to reveal molecular mechanisms underlying nectar synthesis and secretion. Fang et al. (2013) detected 2405 DEGs by incorporating multi-level microarray data of SWT (Si-Wu-Tang, a traditional Chinese medicine). Moreover, they found that 20 proteins targeted by SWT were encoded by these DEGs and could be targeted by two FDA-approved drugs and 39 experimental drugs, which elaborated the potential pharmacological mechanisms of SWT. In this study, we identified a total of 35,327 unigenes differentially expressed between leaf and flower tissues: 26,680 up-regulated and 8647 down-regulated. Notably, we found three important genes, *SK1, C4H*, and *HCT*, were up-regulated in flowers (Figure 2). SK1, C4H, and HCT are key enzymes involved in the synthesis pathway of chlorogenic acid, which is one biomarker used by the Chinese Pharmacopoeia for evaluating the quality of *L. japonica*. This result suggested the content of chlorogenic acid was higher in *L. japonica* flowers than leaves. Previous study reported that *L. japonica* flower buds contained a higher proportion of chlorogenic acid than flowers (Geng et al., 2005). Thus, this study will broaden our understanding of the distribution of chlorogenic acid in different *L. japonica* tissues.

TFs are important DNA-binding proteins. TFs affect the access of RNA polymerase to the gene promoter (Udvardi et al., 2007) and play an important role in the regulation process of gene transcription (Alves et al., 2013) by interacting with other components of the transcriptional machinery. Here, a total of 144 potential TF unigenes were identified by comparing *L. japonica* unigenes with the PlnTFDB. Among them, the main TF families include MYB, C2H2, and ERF. For instance, the MYB family was reported to be the largest TF family in *Arabidopsis* (Yanhui et al., 2006) and could be involved in various biological processes such as regulation of stress responses. Although TFs play important roles in regulating almost each aspect of the organism's metabolism, understanding how the TF-based regulation network eventually affects phenotypes remains difficult. A TF-based regulation network in *L. japonica* was thus constructed, in which *ein3* and *gbf1* were up-regulated, while *nap* was down-regulated in leaves compared with flowers. This result indicated that some TFs have special distributions in plant tissues, which may be linked with their regulatory roles in metabolic activity in *L. japonica*.

## Conclusions

This study characterized the transcriptome profiles in the leaves and flowers of *L. japonica* using NGS technology. A total of 35,327 DEGs were identified between leaves and flowers. Among them, 6602 DEGs were assigned within some important biological processes including “Metabolic process,” “Response to stimulus,” and “Cellular process.” KEGG analysis showed that three possible enzymes involved in the biosynthesis of chlorogenic acid were up-regulated in flowers, which revealed new insight into the molecular regulation of chlorogenic acid metabolism in the flower tissues. Meanwhile, the TF-based regulation network in *L. japonica* showed three DEGs for TFs between leaves and flowers, suggesting their regulatory roles in metabolic activity in *L. japonica*. Overall, this study provided a global picture of different gene expression patterns between leaves and flowers in *L. japonica*. These results not only provide a useful genomic resource of *L. japonica* but will also shed light on functional genomics research on *L. japonica* in the future.

## Author contributions

ML initiated and designed the research. LZ, YL, CF performed and analyzed the experiments. JX, JG, GW, and HJ Contributed reagents/materials/analysis tools. LZ wrote the paper. YL and ML revised the paper.

### Conflict of interest statement

The authors declare that the research was conducted in the absence of any commercial or financial relationships that could be construed as a potential conflict of interest.
