# Supplementary figures and images for: Different Gene Expression Patterns between Leaves and Flowers in Lonicera japonica Revealed by Transcriptome Analysis
Source: Front Plant Sci. 2016 May 10;7:637. doi: 10.3389/fpls.2016.00637 (PMC4861853; doi:10.3389/fpls.2016.00637)

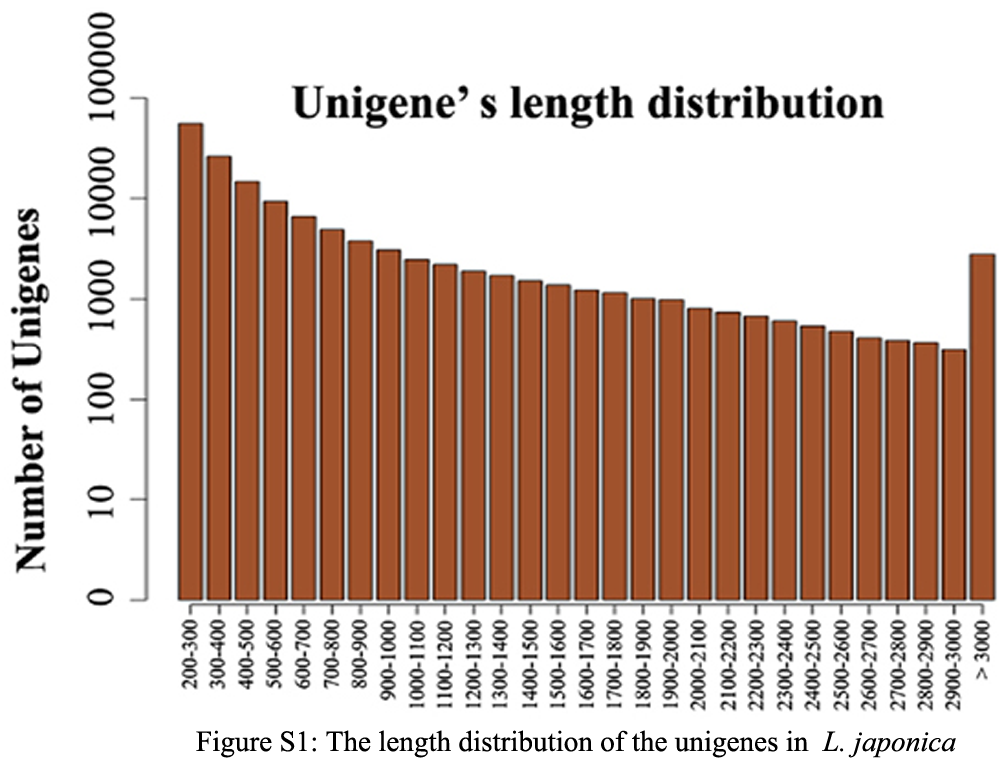

Supplement: Supplementary file 6 [file Image1.tif]

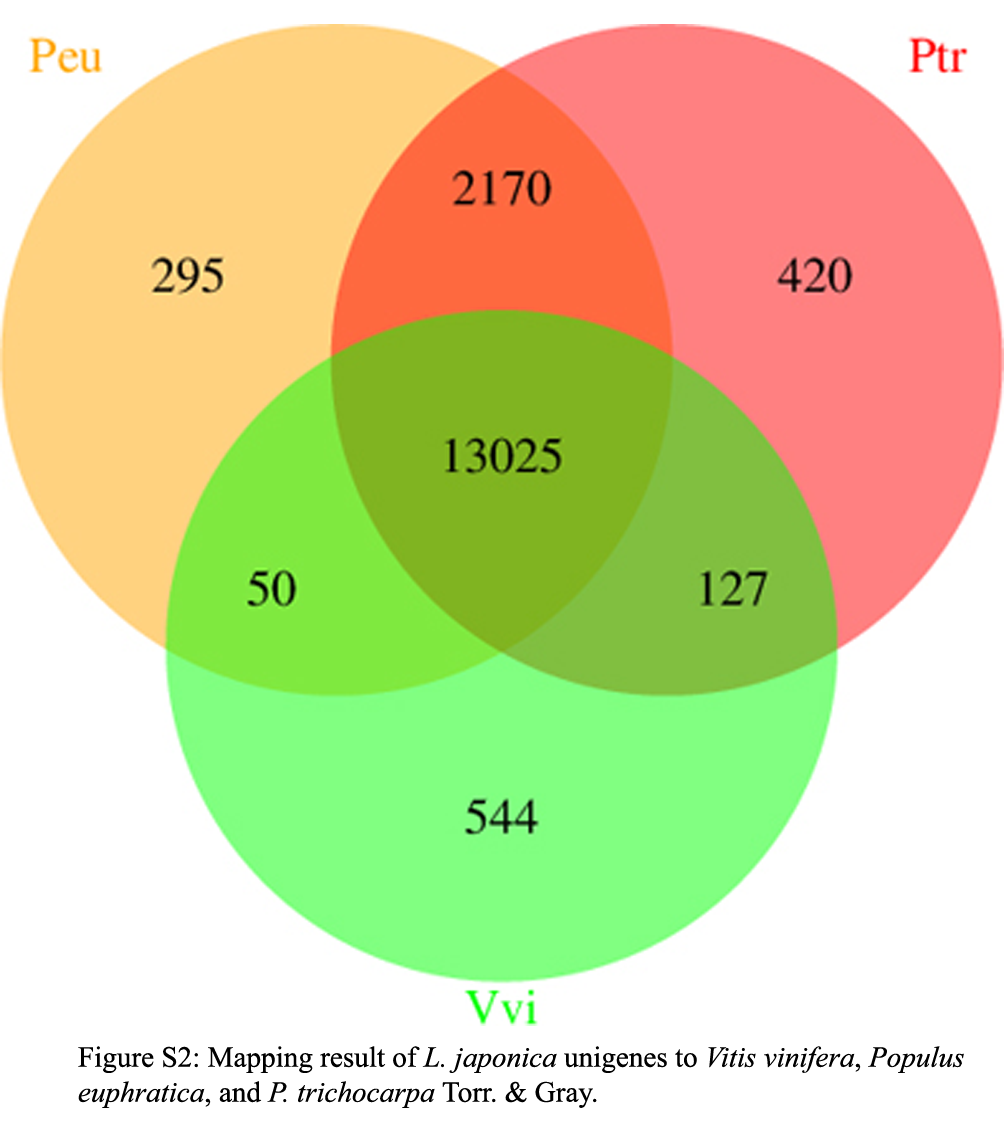

Supplement: Supplementary file 7 [file Image2.tif]

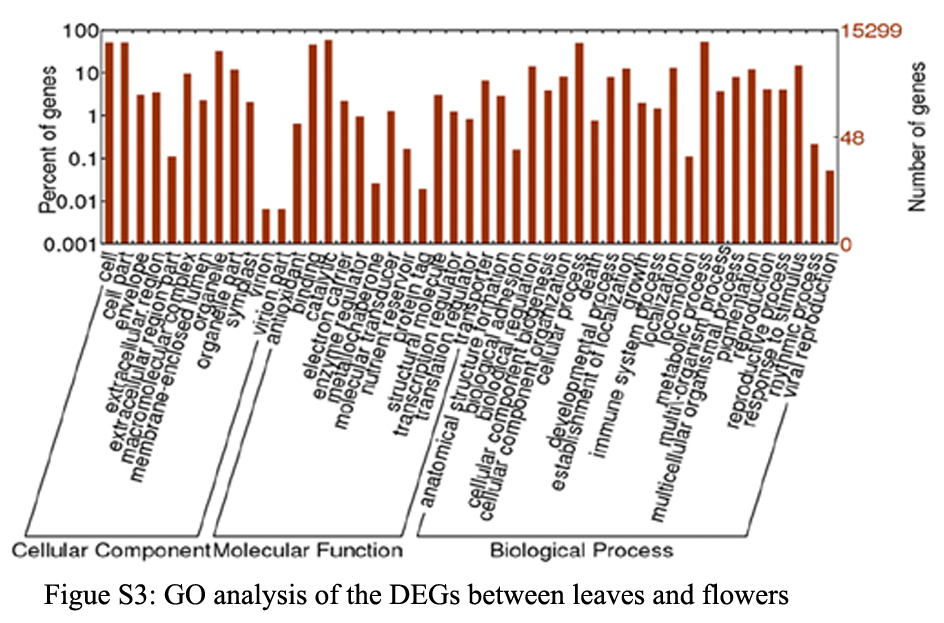

Supplement: Supplementary file 8 [file Image3.tif]

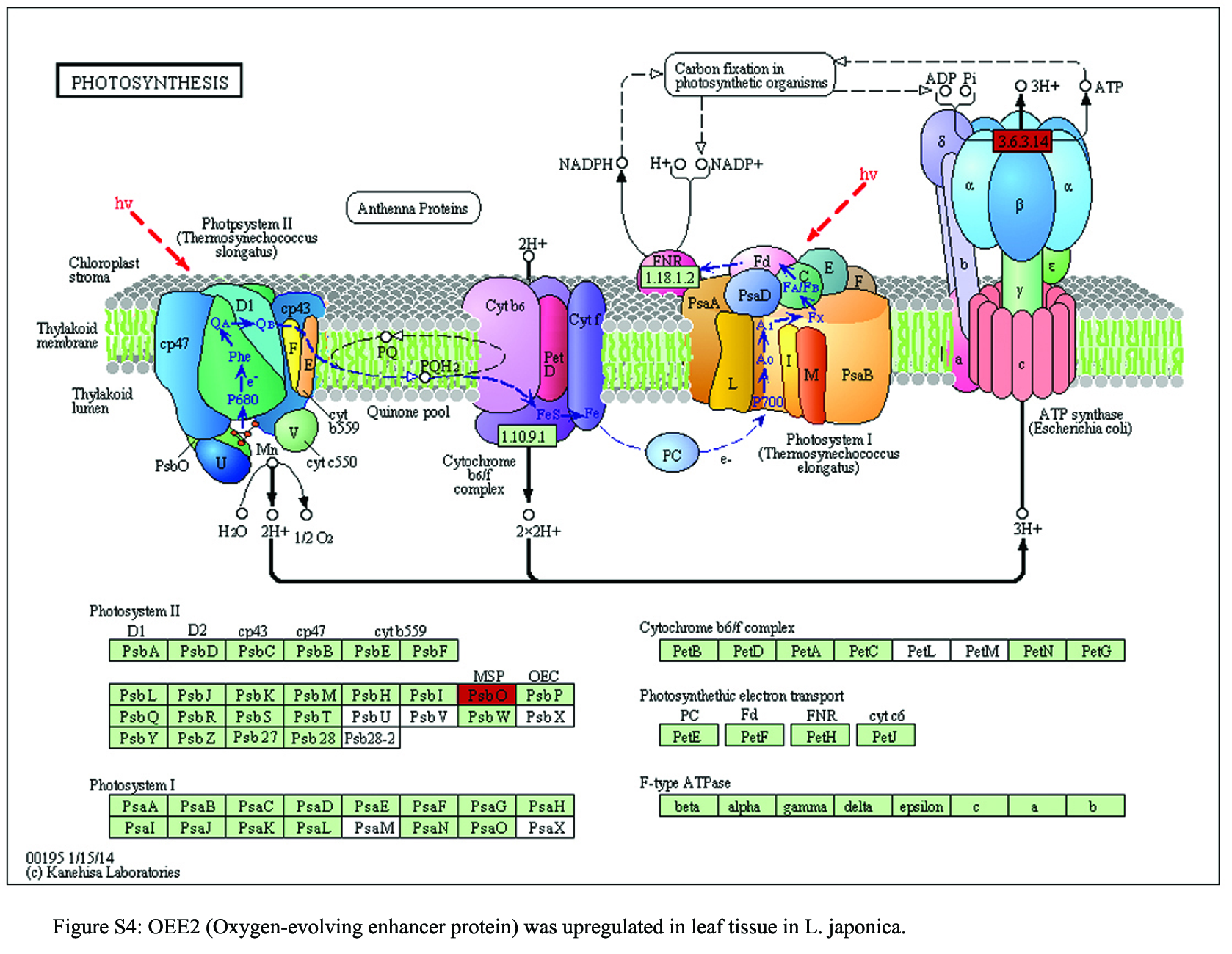

Supplement: Supplementary file 9 [file Image4.tif]

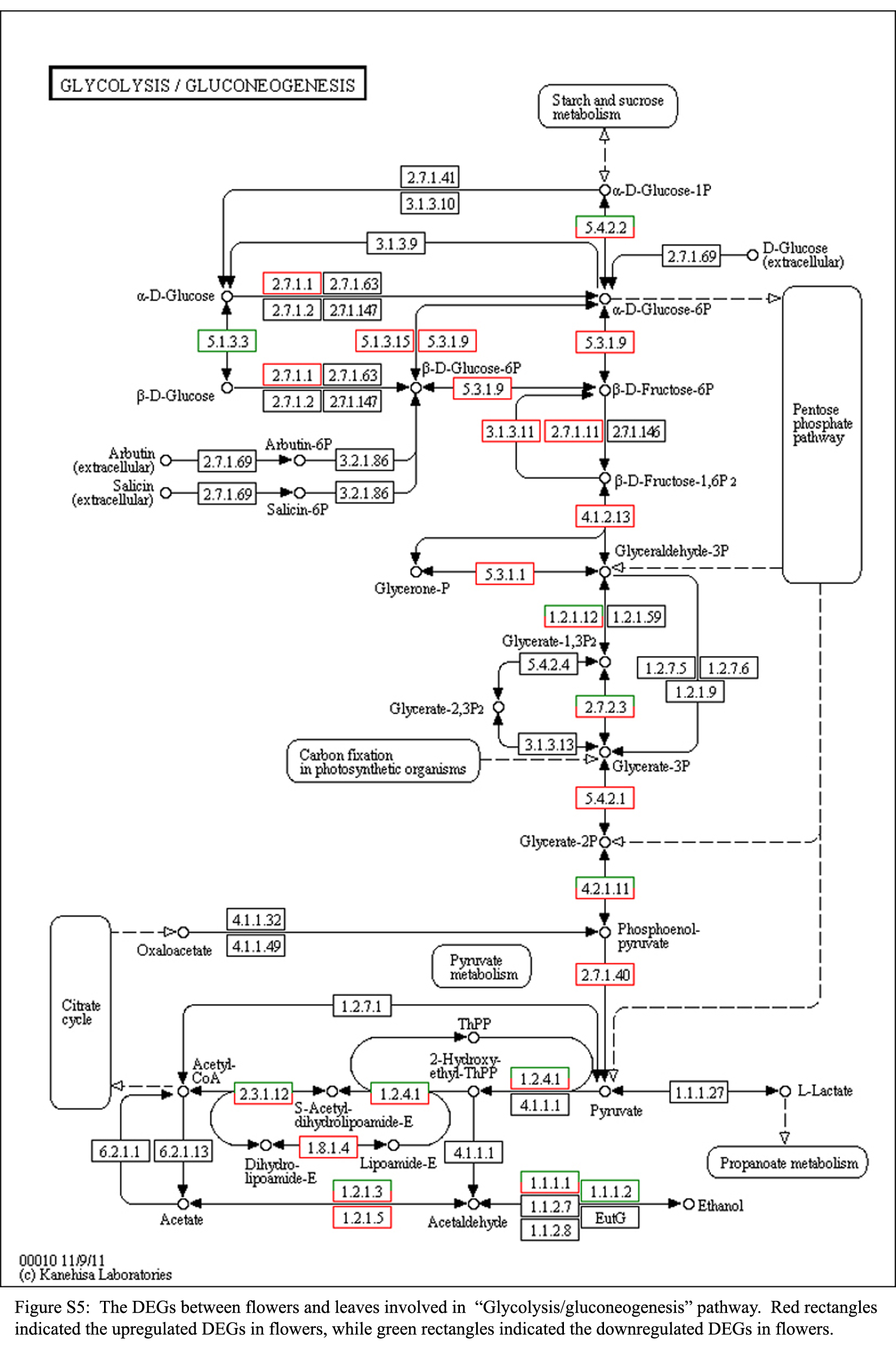

Supplement: Supplementary file 10 [file Image5.tif]

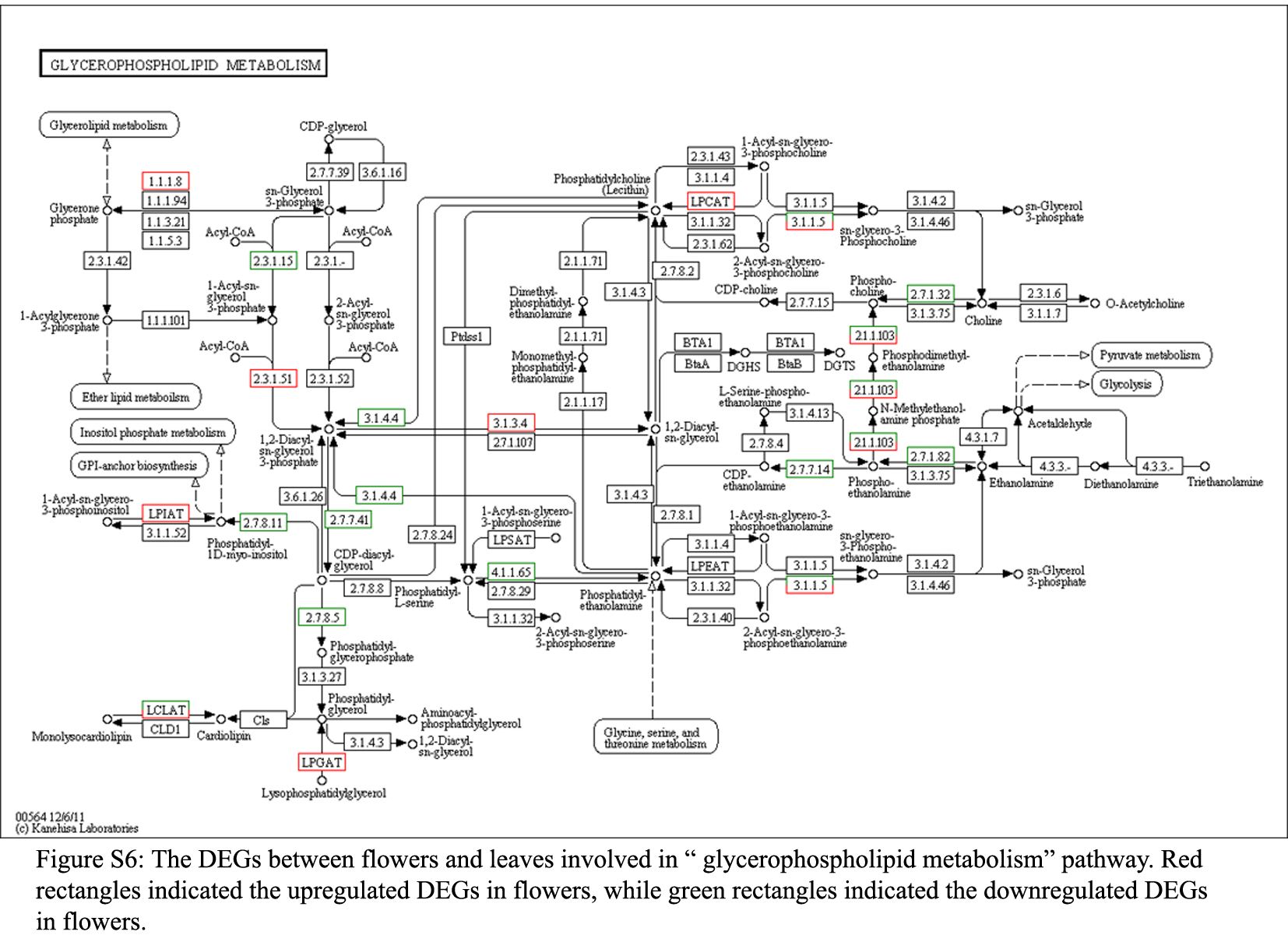

Supplement: Supplementary file 11 [file Image6.tif]
